# Mechanisms governing the accessibility of DNA damage proteins to constitutive heterochromatin

**DOI:** 10.3389/fgene.2022.876862

**Published:** 2022-08-26

**Authors:** Anastasia Roemer, Lanah Mohammed, Hilmar Strickfaden, D. Alan Underhill, Michael J. Hendzel

**Affiliations:** Department of Oncology, Faculty of Medicine and Dentistry, University of Alberta, Edmonton, AB, Canada

**Keywords:** constitutive heterochromatin, accessibility, phase separation, diffusion, cell nucleus, live cell imaging microscopy, DNA damage (DDR), double-strand break (DSB) repair

## Abstract

Chromatin is thought to regulate the accessibility of the underlying DNA sequence to machinery that transcribes and repairs the DNA. Heterochromatin is chromatin that maintains a sufficiently high density of DNA packing to be visible by light microscopy throughout the cell cycle and is thought to be most restrictive to transcription. Several studies have suggested that larger proteins and protein complexes are attenuated in their access to heterochromatin. In addition, heterochromatin domains may be associated with phase separated liquid condensates adding further complexity to the regulation of protein concentration within chromocenters. This provides a solvent environment distinct from the nucleoplasm, and proteins that are not size restricted in accessing this liquid environment may partition between the nucleoplasm and heterochromatin based on relative solubility. In this study, we assessed the accessibility of constitutive heterochromatin in mouse cells, which is organized into large and easily identifiable chromocenters, to fluorescently tagged DNA damage response proteins. We find that proteins larger than the expected 10 nm size limit can access the interior of heterochromatin. We find that the sensor proteins Ku70 and PARP1 enrich in mouse chromocenters. At the same time, MRE11 shows variability within an asynchronous population that ranges from depleted to enriched but is primarily homogeneously distribution between chromocenters and the nucleoplasm. While larger downstream proteins such as ATM, BRCA1, and 53BP1 are commonly depleted in chromocenters, they show a wide range of concentrations, with none being depleted beyond approximately 75%. Contradicting exclusively size-dependent accessibility, many smaller proteins, including EGFP, are also depleted in chromocenters. Our results are consistent with minimal size-dependent selectivity but a distinct solvent environment explaining reduced concentrations of diffusing nucleoplasmic proteins within the volume of the chromocenter.

## Introduction

Pericentric heterochromatin is the region of chromatin juxtaposed to the centromeres and is composed of major satellite repeats (Guenatri et al., 2004). In mouse nuclei, pericentric heterochromatin forms cytologically visible “chromocenters” with DNA stains such as DAPI (Probst & Almouzni, 2008). Chromocenters are epigenetically distinguished by H3K9me3 marks written by the histone methyltransferases SUV39H1 and H2 (Müller-Ott et al., 2014). H3K9me3 marks recruit HP1 proteins, which might facilitate chromatin compaction by dimerizing and/or oligomerizing to bridge nucleosomes (Larson et al., 2017; Machida et al., 2018; Keenen et al., 2021). Pericentric heterochromatin is critical for genome stability, and when disrupted, chromosomal abnormalities, defects in segregation, and increased tumorigenesis are observed in mouse models (Peters et al., 2001; Taddei et al., 2001). Studying the distribution of transcriptional regulators relative to chromatin density using epigenetic modifications to classify chromatin compartments revealed an inverse correlation between chromatin density and protein size, with only the smallest proteins freely accessing heterochromatic regions associated with repressive marks (Miron et al., 2020). Further, Maeshima et al. (2015) investigated the importance of small transcription factor size (about 5 nm) in accessing the condensed interior of topologically associated domains (TADs). They demonstrate in silico that 5 nm spherical objects have free movement in condensed chromatin, 10 nm objects have attenuated movement, and objects larger than 15 nm are excluded entirely (Maeshima et al., 2015). Hihara et al. (2012) similarly used computer simulation to model the movement of EGFP pentamers modelled as 13 nm spheres through nucleosomes modelled as 10 nm spheres. They found that the modelled EGFP pentamers showed attenuated movement and penetration into 10 nm spheres when modelled at high concentrations expected of compact chromatin. Gorisch et al. (2005) used FITC-labelled dextrans of sizes 42, 77, 148, 282, 464, and 2500 kDa dextrans to compare how molecular weight (MW) affects molecule concentration in chromocenters, which is a predicted size range of approximately 6.5–51 nm. They showed reduced access of the dextrans 282 kDa and above in HeLa cell heterochromatin, and this accessibility was increased by increasing chromatin acetylation. An attractive model due to its intuitive simplicity is that size-based accessibility resulting from chromatin compaction restricts availability to the interior (Bancaud et al., 2009). In this model, diffusion into heterochromatin is limited by the size of pores between chromatin fibres or nucleosomes. Size-based accessibility could compromise genomic stability if the sensor proteins MRE11, Ku70/80, and PARP1 exceed this size limit. For example, a complex of Ku70/80 will be 150 kDa (Walker et al., 2001), three times the mass of a typical transcription factor (Maeshima et al., 2015) and has an approximate height and width of 7 and 12 nm (Rivera-Calzada et al., 2007). Thus, it is important to understand if there is significant size-dependence in chromocenter accessibility and, if there is, how this relates to sizes of DNA damage sensing and repair machinery.

DNA double-strand breaks (DSBs) in pericentric heterochromatin are repaired primarily by the homologous repair (HR) pathway or non-homologous end joining (NHEJ) pathway in a cell cycle-dependent manner (Tsouroula et al., 2016). In S and G2, DSBs relocate to the periphery of chromocenters and undergo HR, but in G1, DSBs remain in the chromocenter core and undergo NHEJ (Tsouroula et al., 2016). This suggests that proteins involved in NHEJ repair do not have difficulty accessing the interior of chromocenters. However, chromatin decompaction has been proposed as necessary for DSB repair in heterochromatin (Ayoub et al., 2008; Goodarzi et al., 2009; Noon et al., 2010). The relaxation of chromatin structure in response to DNA double-strand breaks could be a requirement for this accessibility.

Beyond to the potential of molecular size to restrict accessibility to chromocenters, numerous recent studies suggest that chromocenters behave as phase-separated compartments (Larson et al., 2017; Strom et al., 2017, 2021; Larson & Narlikar, 2018; Strickfaden et al., 2019; Wang et al., 2019; Erdel et al., 2020), which could provide an alternative mechanism for reducing the concentration of a diffusing protein below that of the surrounding nucleoplasm. Phase separation is emerging as a mechanism to generate membraneless compartments contributing to the subcellular organization of biomolecules. Phase separation occurs when molecules in a solution capable of multivalent interactions reach a critical concentration and undergo unmixing from the solvent to form a stable microenvironment termed a condensate (Boeynaems et al., 2018). By this mechanism, molecules that favourably interact with the environment of the condensate may enter freely, but molecules with unfavourable interactions will be depleted. Interestingly, some small inert proteins and molecules, including EGFP (Bancaud et al., 2009) and the YFP trimer construct (89 kDa) used by Strom et al. (2017), are depleted from chromocenters relative to the surrounding nucleoplasm despite EGFP being smaller than most transcription factors with a molecular weight (MW) of 27 kDa, a diameter of 2.4 nm, and a length of 4.2 nm (Hink et al., 2000) This is unlikely to be explained simply by differences in density and increased volume exclusion in the chromocenters since the measured density of chromocenters is 208 mg/mL while the surrounding nucleoplasm measures 136 mg/mL (Imai et al., 2017) in living cells.

In this study, we examined the chromocenter concentrations and diffusion of multiple DNA double-strand break sensor, mediator, and effector proteins in living murine cells without DNA damage using fluorescent protein-tagged transfected proteins. This informs us about the ability of proteins involved in sensing and repairing DNA through both the NHEJ and HR pathways to access the interior of chromocenters. We compared the relative nucleoplasmic and chromocenter concentrations and measured diffusion coefficients of selected proteins to determine if accessibility or diffusion rates within chromocenters are directly correlated with apparent molecular weight. We find that there is no clear relationship between molecular weight and the extent of depletion within chromocenters. Nonetheless, DDR proteins did show substantial differences in concentration within chromocenters, and many showed a large range of concentrations within cell populations. Chromatin density alone did not explain the depletion of proteins from chromocenters. When we compared the accessibility of EGFP in living cells with recombinant GFP perfused into fixed cells, only the living cells showed depletion of EGFP relative to the surrounding nucleoplasm. The sensor proteins PARP1 and Ku70 were typically enriched in chromocenters, while MRE11 distribution varied between cells with individual cells found to be enriched, depleted, or evenly distributed across the cell population. Since MRE11 can bind PAR (Haince et al., 2008), we tested the effect of PARP1/2 inhibition and found no impact on the distribution of MRE11, indicating that this was not due to an increase in DNA damage or PAR accumulation in chromocenters. Importantly, all proteins examined showed some ability to access the interior of chromocenters demonstrating that DNA damage-associated chromatin decondensation is not required for large DDR proteins to have access to nuclear heterochromatin domains.

## Materials and methods

### Cell culture

C3H/10 T1/2 cells from ATCC (ATCC CCl-226) were cultured in α-Minimal Essential Medium (Gibco^™^) supplemented with 10% FBS (Gibco^™^) and 1% Penicillin-Streptomycin (Gibco^™^). Cells were maintained in an incubator at 37°C with 5% CO_2_ and humidity. C3H/10 T1/2 cells are a female cell line with fibroblastic morphology in cell culture that was established from 14 to 17 day old C3H mouse embryos but have mesenchymal stem cell-like properties, including the ability to differentiate into distinct cell lineages (Date et al., 2004).

### Transfection

Cells seeded in MatTek dishes were transfected at 60–70% confluency using the Qiagen Effectene transfection kit with some modifications to the protocol. First, 800 ng of DNA was incubated with 3 µL of Enhancer and 100 µL of DNA-condensation buffer (buffer EC) for 15 min rather than the recommended 2–5 min, followed by the addition of 5 µL of Effectene and incubation for 20 min rather than the recommended 5–10. Subsequently, the transfection reagent was added to the cells and cells were left to incubate At 37°C with 5% CO_2_ overnight before imaging the following day.

### Cell imaging

The following day, the transfection medium containing the transfection reagent was replaced with fresh media following a wash step with 1× PBS. Hoechst 33342 was then incubated with the cells at a concentration of 1 µg/mL for 30 min at 37 C to visualize DNA. Next, the medium containing Hoechst 33342 was removed, and cells were washed with 1× PBS before replacement with fresh medium. Fluorescent tagged protein expression in live cells was visualized with a PerkinElmer Ultraview ERS spinning disc confocal microscope equipped with a Hamamatsu Electron Multiplication Charge-Coupled detector device using a 100× 1.4 NA DIC plan-apochromat oil immersion objective lens. In addition, some images were captured using a Leica Falcon SP8 laser scanning confocal microscope with hybrid detectors using an 86× 1.2 NA water plan-apochromat objective lens. Live cell environmental conditions were maintained for both microscopes throughout imaging with a 37°C and CO_2_-controlled live-cell chamber.

### PARPi treatment

For experiments with BMN 673 and ABT 888 PARP1/2 inhibitors, inhibitors were added to cells at a concentration of 10 µM 1 h before imaging and present throughout the experiment.

### Incubation of fixed cells with recombinant EGFP and fluorescent dextrans

CH3/10T1/2 cells were grown to 60–70% confluence and then fixed with 4% paraformaldehyde for 10 min. They were subsequently permeabilized with 0.5% Triton X for 10 min and stained with Hoechst 33342 for 30 min to visualize DNA. Purified Pierce^™^ recombinant GFP protein was diluted to 0.05 µg/µL in 1× PBS and added to cells. They were then allowed to equilibrate for 1 h before imaging by the Leica Falcon SP8 laser scanning confocal microscope with hybrid detectors using an 86× 1.2 NA water plan-apochromat objective lens. FITC labelled 70 kDa (Product no. 46945), and 500 kDa dextrans (Product no. 46947) and TRITC labelled 155 kDa dextrans (Product no. T1287) were obtained from Sigma Aldrich. 70 kDa dextrans were used at a concentration of 0.13 µg/µl in 1× PBS, 155 kDa dextrans at a concentration of 0.29 µg/µL, and 500 kDa dextrans at a concentration of 0.93 µg/µL to keep molarity consistent with the purified GFP. They were then added to cells and allowed to equilibrate for a minimum of 30 min before imaging with the Leica Falcon SP8 laser scanning confocal microscope using an 86× 1.2 NA water plan-apochromat objective lens.

### Quantification of chromocenter partitioning

To capture the relative fluorescent intensity of proteins in the chromocenters relative to the surrounding nucleoplasm, an area within chromocenters was measured for intensity and compared to the intensity of a same-sized area in the nucleoplasm. The intensity was measured using FIJI by creating circular regions of interest and measuring integrated intensity following background subtraction (https://fiji.dc). Analysis was performed in Microsoft Excel 365. Some proteins were observed to form nuclear foci. Cells with foci were more common with increased expression and were excluded from analysis for all proteins with the exception of Rad51 and RNF 168, where foci were present at all expression levels. Consequently, the analyzed cell population was biased towards low protein expression. An *n* of 30 cells was used for the quantification of each protein.

### Fluorescent correlation spectroscopy and diffusion calculation

Fluorescent correlation spectroscopy (FCS) measurements were performed using the Leica Falcon SP8 laser scanning confocal microscope with an 86× 1.2 NA water plan-apochromat objective lens and hybrid detectors. Cells were transfected following the above-described protocol and stained with Hoechst 33342 to visualize DNA. Before imaging, the culture medium was replaced with phenol red-free DMEM to reduce phenol red-derived fluorescence. During FCS measurements, cells were maintained at 37°C and 5% CO_2_ in a live-cell environmental chamber. FCS measurements were collected for 5 s with three repetitions at each spatial point. Curve fitting was calculated with the Leica Application Suite X software with photobleaching correction and spark removal at sensitivity level 20 using the diffusion with triplet model with triplet amplitude set to 0.10 and triplet time set to 0.010 to remove triplets from fitting. A single component fit was optimal for all proteins except for RNF168 and 53BP1, for which a single component or two-component fit appeared equal, so a one-component fit was maintained for consistency. The final diffusion value was calculated from measurements collected on three separate days from at least 30 cells. Unambiguously incorrect measurements were removed from the overall calculation. These occurred when zero or near zero molecules were detected despite detection in subsequent acquisitions at the same location.

Graphs were created in RStudio using the ggplot2 package (Wickham, 2016).

## Results

### PARP1-GFP and Ku70-GFP sensor proteins display enrichment in chromocenters while MRE11-YFP displays heterogenous behavior

**FIGURE 1:**
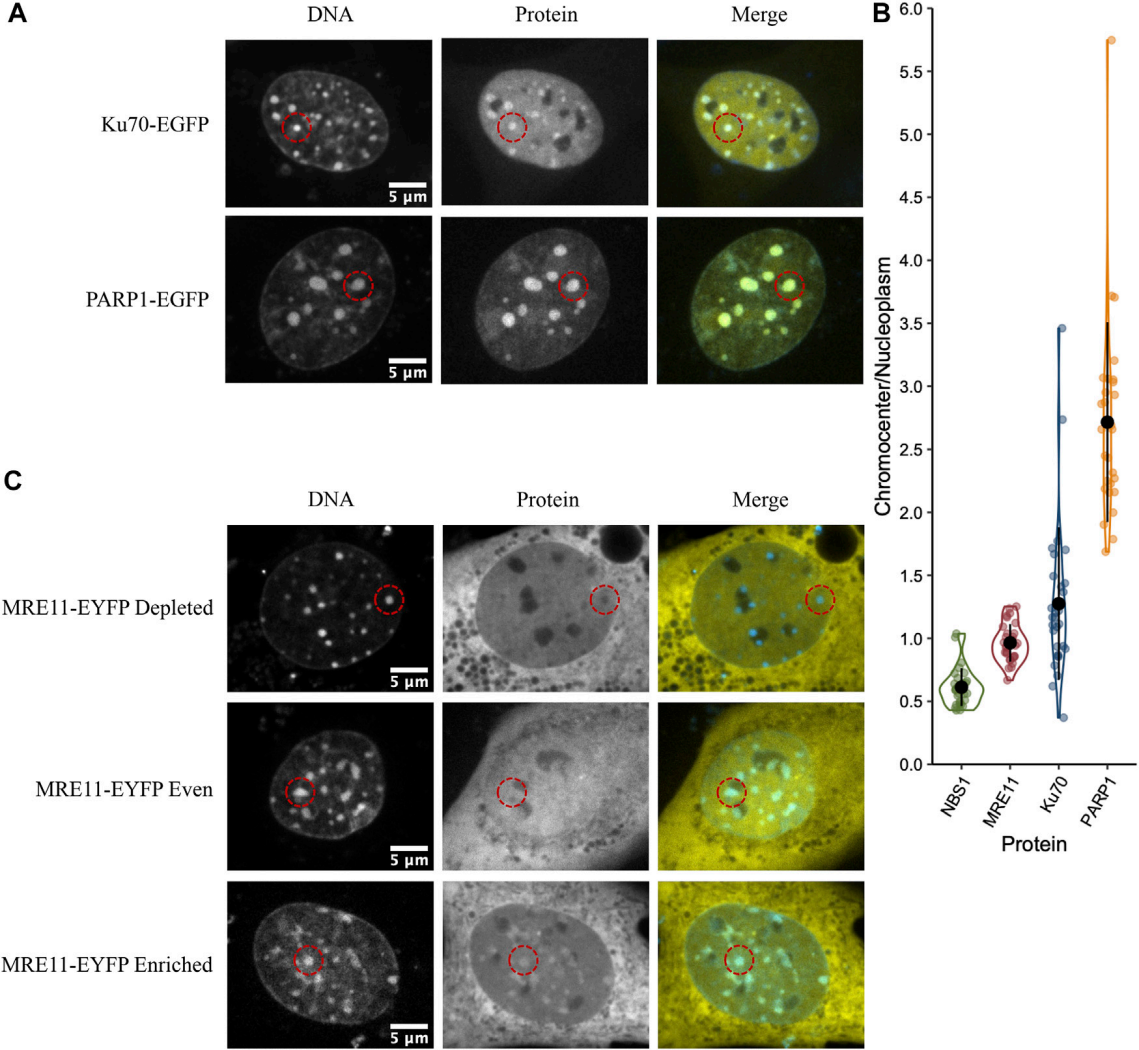
DNA damage sensors PARP1 and Ku70 are enriched in chromocenters while the DNA damage sensor MRE11 displays heterogeneous behaviour. **(A)** CH3/10T1/2 cells were transfected with either PARP1-EGFP or Ku70-EGFP and stained with Hoechst 33342 to visualize DNA. **(B)** Quantification of relative fluorescent protein chromocenter intensity for proteins NBS1-EGFP, MRE11-YFP, Ku70-EGFP, and PARP1-EGFP (*n* = 30 cells for each protein). **(C)** CH3/10T1/2 cells were transfected with MRE11-YFP and stained with Hoechst 33342 to visualize DNA. Depleted, homogenous, and enriched distributions of MRE11-YFP are illustrated.

Poly (ADP-ribose) polymerase 1 (PARP1) and Ku70 (XRCC6) are both sensors of DNA damage. PARP1-catalyzes poly(ADP-ribosyl)ation, which is an initial step in the DNA damage response that facilitates both Ku70/80 recruitment, the initial step in the NHEJ pathway, and MRE11 recruitment, the nuclease that initiates end resection required for the homologous recombination (HR) repair pathway (Rivera-Calzada et al., 2007; Haince et al., 2008; Yu et al., 2012). PARP1, MRE11, and Ku proteins have all been ascribed the role of DNA double-strand break sensor (Ray Chaudhuri and Nussenzweig, 2017; Huang & Zhou, 2020), where they initiate the DNA double-strand break signalling and repair response. As the sensing of double-stranded breaks is essential to their subsequent repair, we wanted to test the ability of these three critical proteins to access the interior of chromocenters. For example, the Ku heterodimer is approximately 12 × 7 × 7 nm (Walker et al., 2001). The GFP tag may increase this to 15 nm or more; hence, Ku should be excluded based upon a 10 nm diameter pore size. To test the accessibility of chromocenters to sensor proteins, mouse cells were transfected with plasmids encoding the protein of interest fused to a fluorescent protein. To visualize the behaviour of transfected proteins, images were collected in living cells, and chromocenter location was determined by staining DNA with Hoechst 33342. Images were collected by spinning disk or laser scanning confocal microscopy. Both PARP1-GFP and Ku70-GFP visually display enrichment in chromocenters, while MRE11-YFP displayed heterogeneous behaviour (Figures 1A,C). Quantification of protein concentration in chromocenters was assessed relative to the nucleoplasm using the integrated intensity of the fluorescent protein tag. PARP1 and Ku70 both showed enrichment in the chromocenters, with PARP1 showing the strongest enrichment (Figure 1B). Ku70 showed subtle depletion in a subset of cells. The ability of Ku70 to enrich in the chromocenter implies that it is not too large to enter chromocenters, where it can then interact with DNA or proteins to accumulate beyond the nucleoplasmic concentration. In the case of MRE11, there are subsets of cells that are either clearly depleted, clearly enriched, or homogeneously distributed (Figure 1C). Interestingly, NBS1, part of the MRE11/NBS1/Rad50 (MRN) complex, is consistently depleted from chromocenters (Figure 1B).

**FIGURE 2:**
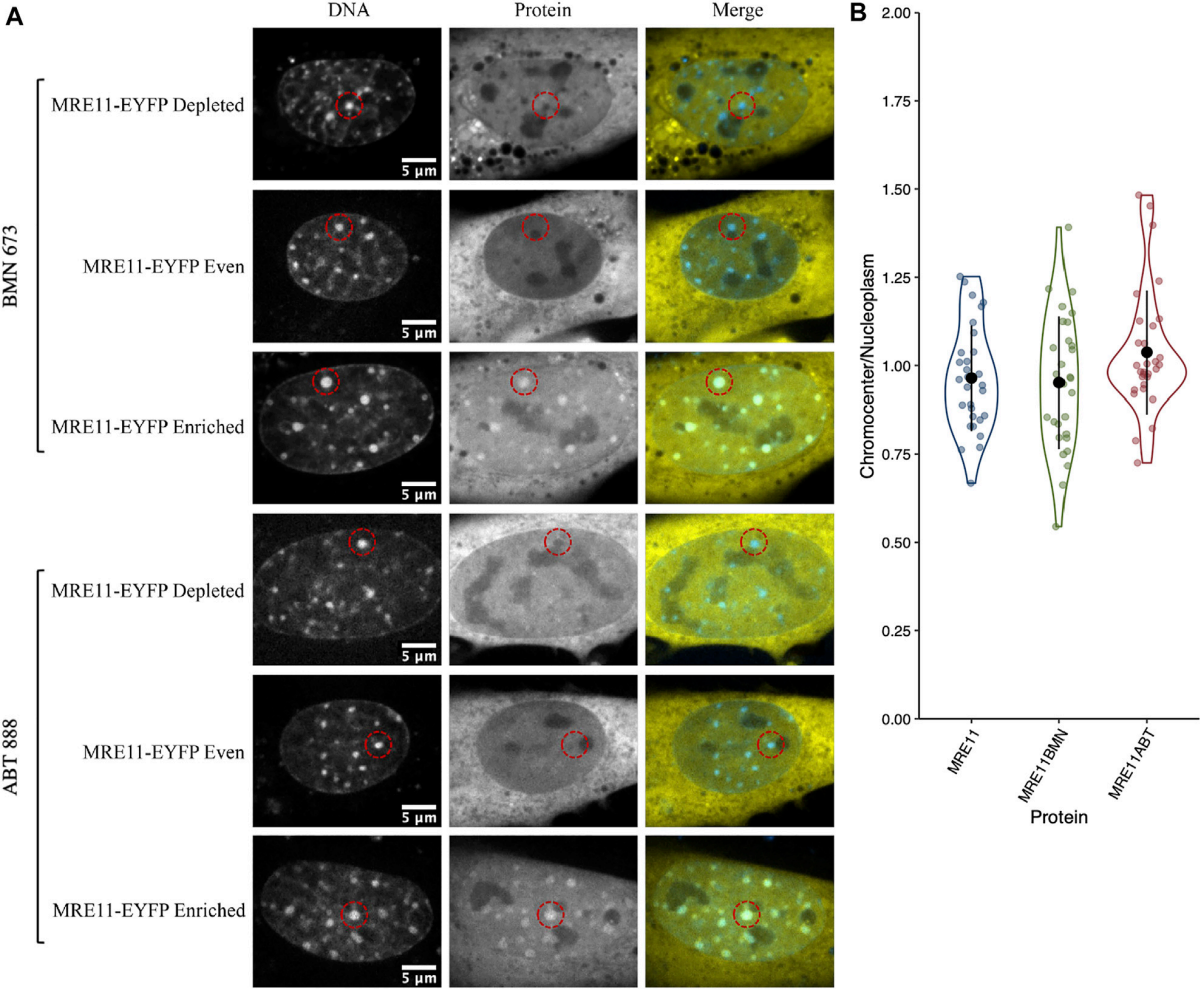
MRE11-YFP chromocenter heterogeneity is not dependent on poly (ADP-ribosyl)ation **(A)** CH3/10T1/2 cells were transfected with MRE11-YFP, Hoechst 3 3342 was added to visualize DNA, and then incubated with either BMN 673 or ABT 888 at a concentration of 10 µM for 1 h before imaging. An example of depleted, homogenous, and enriched distributions are provided for both inhibitor groups **(B)** Graph shows quantification of relative chromocenter intensity for each group. MRE11-YFP control is included for direct comparison. Each protein has *n* = 30. MRE11-YFP control is not significantly different from either MRE11-YFP treated with BMN 673 (*p* = 0.77) or MRE11-YFP treated with ABT 888 (*p* = 0.087).

We previously demonstrated that MRE11 binds to poly(ADP-ribose)(PAR) and is responsible for the rapid recruitment of MRE11 to sites of DNA damage (Haince et al., 2008). Based on the accumulation of PARP1 in chromocenters, we tested if MRE11-YFP enrichment in chromocenters is due to PARylation within chromocenters. The PARP1 and 2 inhibitors BMN 673 (Talazoparib, 10 µM) or ABT 888 (Veliparib, 10 µM) were incubated with cells for 1 h before imaging (Krietsch et al., 2012; Caron et al., 2019). Both inhibit PARP1 and 2 catalysis of poly (ADP-ribosyl)ation (PARylation) (Donawho et al., 2007; Shen et al., 2013). Neither BMN 673 nor ABT 888 prevented the accumulation of MRE11 in chromocenters nor affected the heterogeneous distribution of MRE11-YFP across the cell population (Figures 2A,B). Since PARP1, Ku70, and MRE11 all show the ability to enter into chromocenters, the results are inconsistent with a 10 nm exclusion limit, and size-based exclusion appears not to be a limitation to the sensing of DNA double-strand breaks in heterochromatin.

### Mediator and effector proteins show heterogeneous accessibility that does not correlate with HR or NHEJ pathway involvement

**FIGURE 3:**
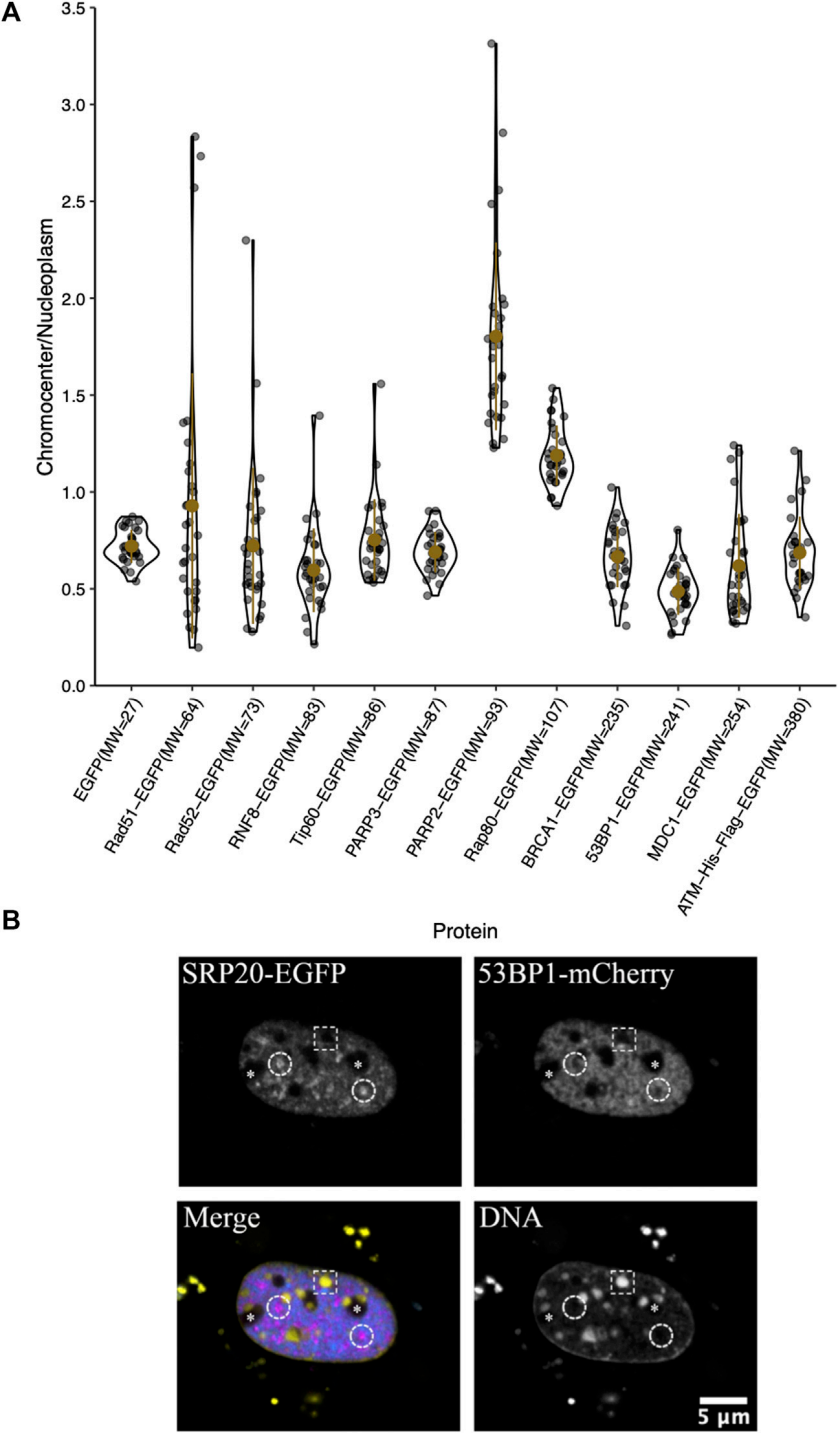
Chromocenter localization of mediator and effector proteins. **(A)** Graph showing quantification of relative chromocenter intensity for mediator and effector proteins. Cells were transfected with a plasmid encoding the protein of interest and stained with Hoechst 33342 to visualize DNA (*n* = 30 for each protein). **(B)** A cell expressing the splicing factor SRp20-EGFP and 53BP1-mCherry and counterstained with Hoechst 33342 to contrast the chromatin. The dashed circles indicate the positions of splicing factor compartments while the dashed rectangle illustrates the position of a chromocenter. Asterisks indicate the positions of nucleoli.

The large downstream mediators of DNA damage repair could rely on changes in chromatin accessibility as a result of early chromatin remodelling events (Poirier et al., 1982; Goodarzi et al., 2008, 2009; Noon et al., 2010; Rack et al., 2021). Consequently, we wanted to test their ability to diffuse into the interior of chromocenters in the absence of DNA damage and the associated chromatin remodelling. Transiently expressed proteins were assessed for their relative concentration in chromocenters (Table 1 and Figure 3). For proteins below approximately 200 kDa in size, there does not appear to be any relationship with accessibility (Table 1 and Figure 3). Notably, most of these proteins show depletion in mouse chromocenters, suggesting that some concentration regulation is taking place. In this respect, the depletion of EGFP alone is salient. BRCA1 and PARP3 have a very similar depletion to EGFP despite BRCA1 being, for example, almost 10X the mass of EGFP alone.

**TABLE 1:** Accessibility of DNA damage mediators to chromocenters.

Protein/Dye name	Size (kDa)	Chromocenter status in MEF cells (visual appearance)	Measured chromocenter concentration relative to the nucleoplasm (Min, Max)	Foci formation	Foci publications	Protein dimensions from alpha fold structures (Å) (Jumper et al., 2021) [uniprot accession]
EGFP	27	Depleted	0.79 (0.54, 0.87)	No	N/A	35.47, 41.70, 57.41 [P42212]
Rad51-EGFP	64	Even	0.93 (0.20, 2.8)	short rods	(Tarsounas et al., 2003; Galkin et al., 2005)	103.94, 63.64, 45.69 [Q06609]
Rad52-EGFP	73	Depleted	0.72 (0.28, 2.3)	No	N/A	82.20, 111.39, 134.04 [P43351]
RNF8-EGFP	83	Depleted	0.76 (0.21, 3.7)	No	N/A	205.89, 87.54, 63.38 [O76064]
Tip60-EGFP	86	Depleted	0.75 (0.53, 1.6)	Yes	Wu et al. (2009)	91.57, 86.88, 64.58 [Q92993]
PARP3-EGFP	87	Depleted	0.69 (0.46, 0.90)	No	N/A	103.68, 99.78, 60.30 [Q9Y6F1]
RNF 168-EGFP	92	Enriched	7.0 (1.4, 12)	Yes		177.85, 130.39, 97.67 [Q8IYW5]
PARP2-EGFP	93	Enriched	1.8 (1.2, 3.3)	No	N/A	101.61, 95.84, 83.60 [Q9UGN5]
Rap80-EGFP	107	Enriched	1.2 (0.93, 1.5)	Yes	Soo Lee et al. (2016)	176.16, 135.35, 119.35 [Q96RL1]
NBS1-EGFP	112	Depleted	0.61 (0.43, 1.0)	Yes		123.30, 136.58, 148.48 [O60934]
BRCA1-EGFP	235	Depleted	0.66 (0.31, 1.0)	No	N/A	179.77, 169.90, 181.71 [P38398]
53BP1-EGFP	241	Depleted	0.49 (0.26, 0.80)	Yes	(Kilic et al., 2019; Lukas et al., 2011)	155.69, 163.43, 188.04 [Q12888]
MDC1-EGFP	254	Depleted	0.62 (0.32, 1.2)	Yes		155.48, 181.29, 223.16 [Q14676]
ATM-His-Flag-EGFP	∼380	Depleted	0.67 (0.35, 1.2)	No	N/A	82.45, 116.01, 212.66
						*From PDBe 6K9K (Xiao et al., 2019)

There may be a reduction in the space available for the diffusion of the largest proteins studied. BRCA1-GFP (235 kDa), 53BP1-GFP (241 kDa), MDC1-GFP (254 kDa), and ATM-His-Flag-GFP (∼380 kDa) are all depleted from chromocenters (Table 1 and Figure 3). The most striking was 53BP1, which did not show any examples of accumulation within chromocenters. It was also distinctive for a second reason—its appearance in nuclei revealed additional regions of depletion that corresponded to DNA depleted regions of the nucleus outside of the nucleolus. Expression of a fluorescently tagged splicing factor (SRp20) revealed these to be splicing factor compartments (Figure 3B). 53BP1 has been reported to undergo phase separation *in vitro* and is proposed to participate in forming phase separated compartments surrounding DNA double-strand breaks in cells (Kilic et al., 2019). This could confer poor solubility in liquid compartments that differ from the surrounding nucleoplasm. The nucleolus is also clearly depleted in 53BP1 and has a distinct liquid environment (Figure 3B) (Brangwynne et al., 2011; Feric et al., 2016; Frottin et al., 2019; Lafontaine et al., 2021). We also used the program CIDER to analyze our mediator proteins. We found no obvious differences between them except that 53BP1 has a lower ratio of positively charged residues to negatively charged residues (data not shown) (Holehouse et al., 2015). Notably, except for 53BP1, all DNA damage response proteins have cells within the population that show a near homogeneous distribution between nucleoplasm and chromocenter or examples of cells with protein enrichment in the chromocenter. These results indicate that none of these proteins are too large to enter into chromocenters. However, the large range of relative chromocenter concentrations observed for most of these proteins and the reduced concentration of EGFP in chromocenters suggest mechanisms beyond size-dependent filtering and excluded volume effects reduce the concentrations of freely diffusing nucleoplasmic proteins within the chromocenter volume.

### Diffusion properties of example DNA damage response proteins

**FIGURE 4:**
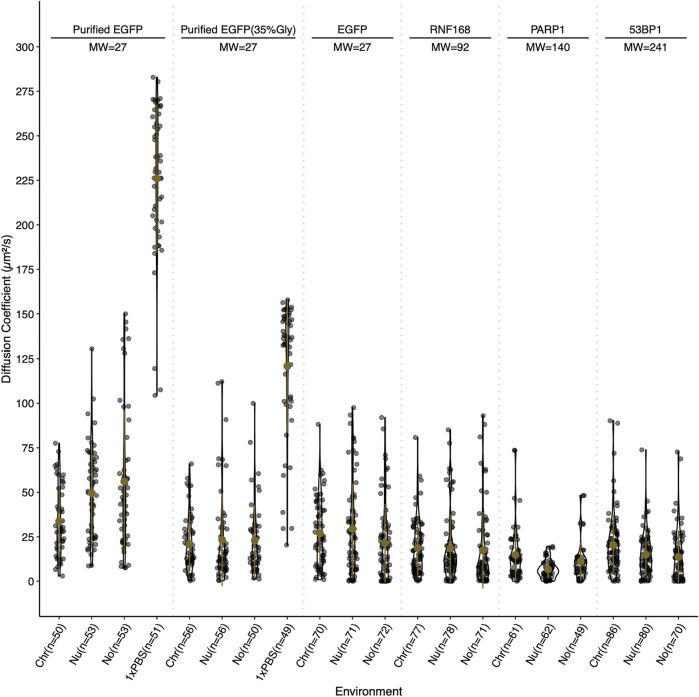
Relative diffusion of EGFP and selected DNA damage response proteins. Diffusion coefficients for EGFP, RNF168-EGFP, PARP1-EGFP, and 53BP1-EGFP in live cells, and purified EGFP in 0 and 35% glycerol in fixed cells. Diffusion coefficients are reported in µm^2^/s and were determined by fluorescent correlation spectroscopy. Diffusion coefficients are reported for the chromocenter (Chr), the nucleoplasm (Nu), the nucleolus (No), and for purified EGFP in 1× PBS (1×PBS). Live cell measurements were gathered at 37°C and fixed cell measurements were collected at 18°C. Both live and fixed cells were stained with Hoechst 33342 to visualize DNA.

Slower diffusion through chromocenters with increasing sizes of EGFP multimers has been proposed to reflect dependence on size (Baum et al., 2014). For globular proteins in solution, an eight-fold increase in mass is predicted to decrease the diffusion rate two-fold. To test whether we observe size-dependent diffusion with the DNA damage proteins, we performed fluorescent correlation spectroscopy (FCS) across a size range with EGFP, RNF168-EGFP, PARP1-EGFP, 53BP1-EGFP, and purified GFP perfused through fixed cells in 1× PBS and 35% glycerol PBS. Values in the nucleolus were also measured and reported. In fixed cells, the calculated diffusion coefficients reflect the density of chromocenters compared to the nucleoplasm, as the average diffusion coefficient for EGFP in chromocenters is 34 μm^2^/s. In contrast, in the nucleoplasm, it is 50 μm^2^/s. However, EGFP in live cells reveals no significant difference, with the chromocenter having a mean diffusion coefficient of 27 μm^2^/s and the nucleoplasm having 29 μm^2^/s. We tested purified GFP diffusion in 35% glycerol because of a previous report that in media containing 40% glycerol, the rotational diffusion of GFP in solution is similar to that of GFP in live cells (Erdel et al., 2020). We found that after the addition of glycerol, the mean diffusion coefficient in the solution was reduced from 226 to 121 μm^2^/s. The diffusion coefficient in chromocenters began to more closely resemble that in living cells at 21 μm^2^/s in 35% glycerol and 34 μm^2^/s in 1× PBS. This is consistent with the viscosity of the nucleoplasm being approximately equivalent to 30–40% glycerol. While EGFP showed an expected mobility reduction in chromocenters relative to the nucleoplasm, RNF168-EGFP, PARP1-EGFP, and 53BP1-EGFP FCS all measured a faster mean diffusion coefficient in the chromocenters compared to the nucleoplasm (Figure 4). RNF168-EGFP and 53BP1-EGFP had similar behaviour, with a mean diffusion coefficient of 19 μm^2^/s in chromocenters for RNF168-EGFP and 20 μm^2^/s for 53BP1-EGFP, and mean nucleoplasmic diffusion coefficients of 19 and 15 μm^2^/s, respectively (Figure 4). These are about two-fold slower than EGFP but correspond to almost four-fold (RNF168-EGFP) and almost 10-fold (53BP1-EGFP) difference in mass. This is consistent with the free diffusion of 53BP1-EGFP monomers but suggests that RNF168 may form a larger diffusing complex than predicted by its molecular weight as a monomer. Interestingly, PARP1-EGFP had a mean diffusion coefficient of 15 μm^2^/s, which is about twice the mean diffusion coefficient measured in the nucleoplasm of 6.7 μm^2^/s (Figure 4). One potential explanation for an unexpectedly higher diffusion rate inside chromocenters is that these proteins are diffusing along the chromatin fibre within chromocenters rather than undertaking 3D diffusion in the associated liquid phase. A second explanation is that these molecules diffuse as larger complexes in the nucleoplasm.

### Non-specific DNA binding may contribute to the abundance of large proteins in chromocenters

**FIGURE 5:**
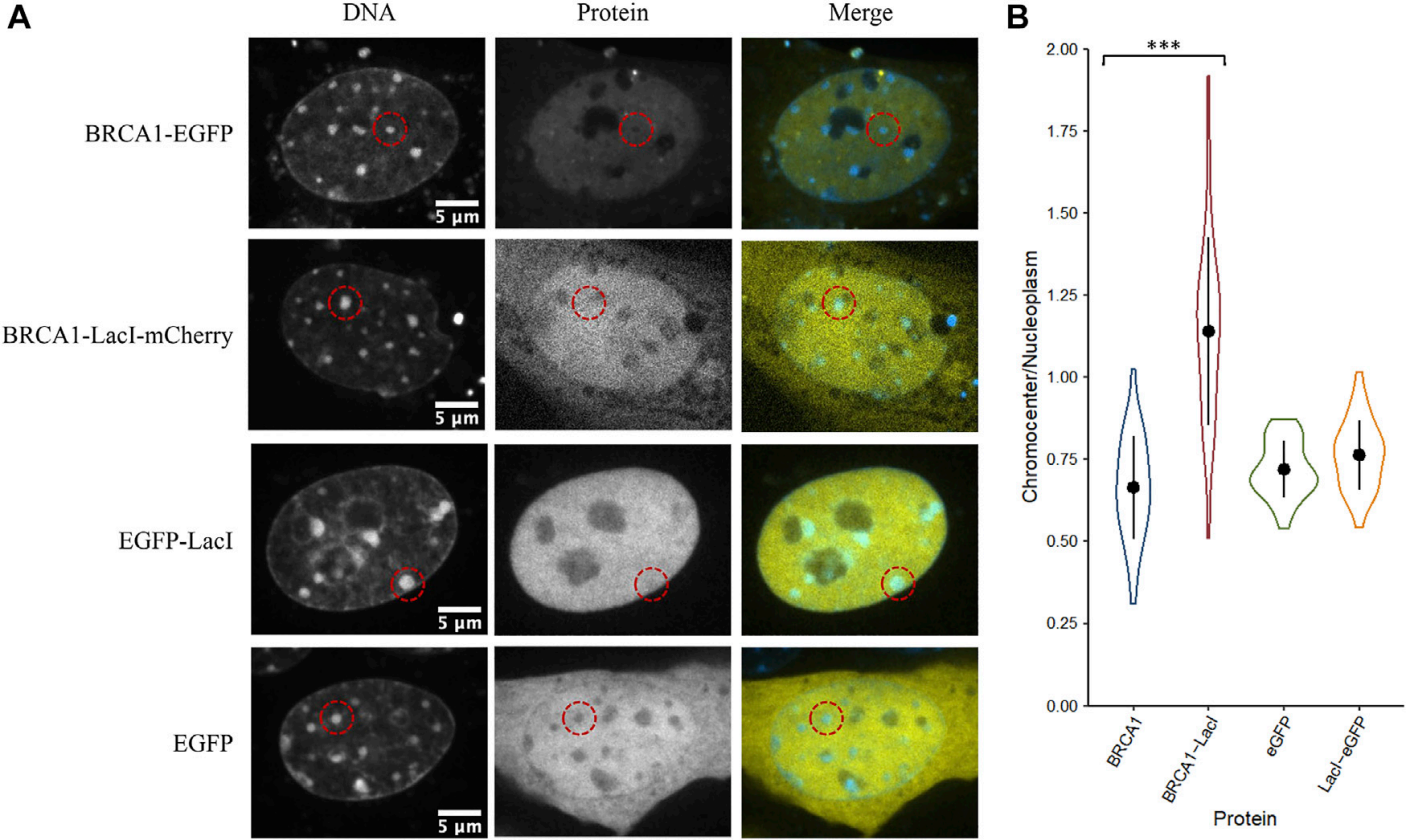
The LacI DNA binding domain may alter the ability of BRCA1 to access chromocenters. **(A)** CH3/10T1/2 cells were stained with Hoechst 33342 to visualize DNA and transfected with plasmids encoding either BRCA1-GFP, BRCA1-LacI-mCherry, LacI-EGFP, or EGFP. **(B)** Quantification of relative chromocenter intensity for each protein. BRCA1-EGFP is significantly different from BRCA1-LacI-mCherry (*p* = 1.3E−09). EGFP is not significantly different from LacI-EGFP (*p* = 0.088) (*n* = 30 for each protein).

The initial results indicate that even large proteins can diffuse into the interior of chromocenters. Collombet et al. (2021) examined the accessibility of the inactive X chromosome territory to RNA polymerase II using single-molecule tracking methods. They showed that RNA polymerase II could freely diffuse into and through the inactive X chromosome territory. The principal difference explaining its depletion within the inactive X chromosome territory is the absence of binding to chromatin within the inactive X territory. Therefore, we wondered if the depletion of proteins we observed resulted from a failure to be retained in chromocenters rather than depletion by barriers to diffusion that prevented entry. We tested a fusion protein of BRCA1 with the LacI DNA binding domain (274 kDa). The specific DNA binding site for LacI is not natively endogenous in the mammalian genome resulting in LacI being unable to bind DNA specifically, yet LacI is known to have non-specific DNA binding to search the genome for its target sequence (Kao-Huang et al., 1977; Hammar et al., 2012; Stracy et al., 2021). Interestingly, we found that the fusion of the LacI DNA binding domain to BRCA1 increased its concentration in chromocenters. As previously described, BRCA1-GFP is depleted from chromocenters with a mean concentration of 66% of the nucleoplasm, whereas BRCA1-LacI-mCherry was enriched to 120% of the nucleoplasmic concentration (Figures 5A,B). This is consistent with non-specific DNA binding contributing to the accumulation of BRCA1 in mouse chromocenters. To further test this, we examined the fusion of the LacI DNA binding domain to EGFP. In contrast to the BRCA1 fusion, however, the fusion of the LacI DNA binding domain to EGFP did not result in a significant difference in EGFP accumulation in chromocenters. Both forms of the protein were depleted relative to the surrounding nucleoplasm (Figures 5A,B). The result with the EGFP fusion suggests that DNA binding is not a determinant of EGFP distribution but may contribute to BRCA1 distribution. One possible explanation for this is that modelling two DNA binding domains joined by a flexible linker predicts enhanced affinity for DNA over those of the two individual domains because both can interact with the DNA (Zhou, 2001). BRCA1 also binds DNA (Paull et al., 2001; Simons et al., 2006) and combined with the LacI domain, synergy in binding could explain the differences between the LacI fusion with EGFP versus BRCA1-mCherry.

### Depletion of EGFP and dextrans cannot be explained by chromatin-mediated volume exclusion

**FIGURE 6:**
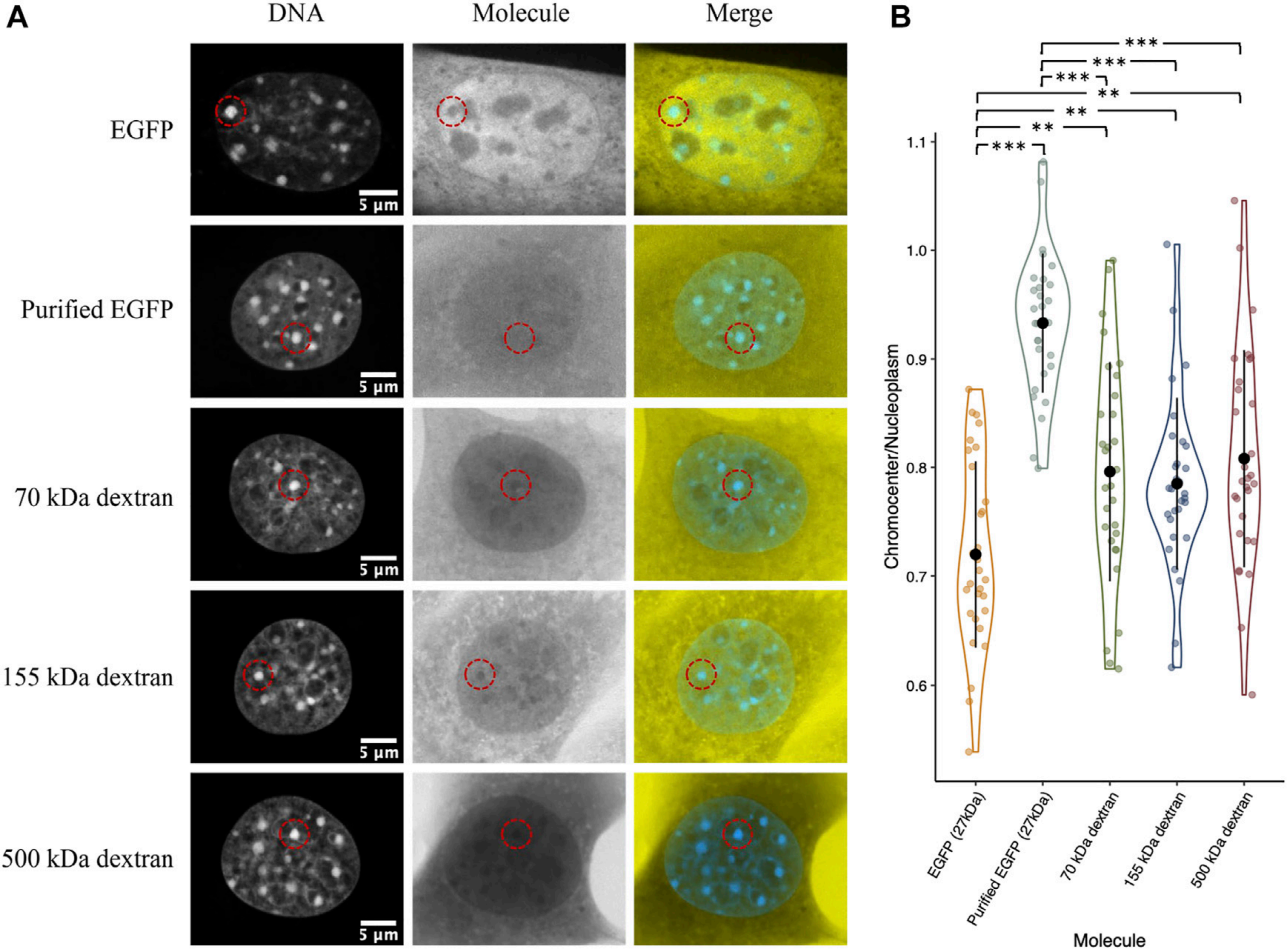
Distribution of purified recombinant GFP and fluorescent dextrans in fixed permeabilized cells. **(A)** CH3/10T1/2 cells were either fixed with paraformaldehyde and permeabilized with Triton X followed by perfusion with purified EGFP or the indicated dextrans diluted in 1× PBS. For comparison, a cell transfected with a plasmid encoding EGFP and imaged live is also shown. Both groups were stained with Hoechst 33342 for DNA visualization. **(B)** Quantification of chromocenter concentration relative to the surrounding nucleoplasm.

Compaction of chromatin into heterochromatin domains logically results in an increased excluded volume since the chromatin must occupy some of the available space (Bancaud et al., 2009). Therefore, we wanted to test if the depletion of inert molecules such as EGFP from chromocenters could be explained simply by a reduction in available space from increased chromatin occupancy. We placed paraformaldehyde-fixed permeabilized CH3/10T1/2 cells with purified GFP in 1× PBS and compared chromocenter partitioning to live cells transiently expressing EGFP. We observed a clear difference between the two groups, with purified GFP having a near homogeneous distribution across the nucleoplasm (Figures 6A,B). We repeated the experiment with 70, 155, and 500 kDa dextrans to further validate this result. Above 2 kDa, dextrans behave as random coils in solution, allowing the prediction of molecular dimensions based on the radius of gyration (R_G_) (Basedow & Ebert, 1979), which is about 8.5, 12.7, and 22.8 nm for the 70, 155, and 500 kDa dextrans, respectively (Oliver & Deen, 1994). Interestingly, despite the large variation in size, there is no significant difference between the dextrans, with each being around 20% depleted from chromocenters relative to the nucleoplasm (Figures 6A,B). Notably, the dextrans are significantly less depleted than EGFP concentrations in chromocenters of living cells, despite the smallest dextran being approximately twice the size of EGFP (Figures 6A,B). This result demonstrates that the observed depletion of proteins from chromocenters cannot be explained solely by volume exclusion effects.

### Spontaneous nuclear focus formation upon expressing DNA damage response proteins

**FIGURE 7:**
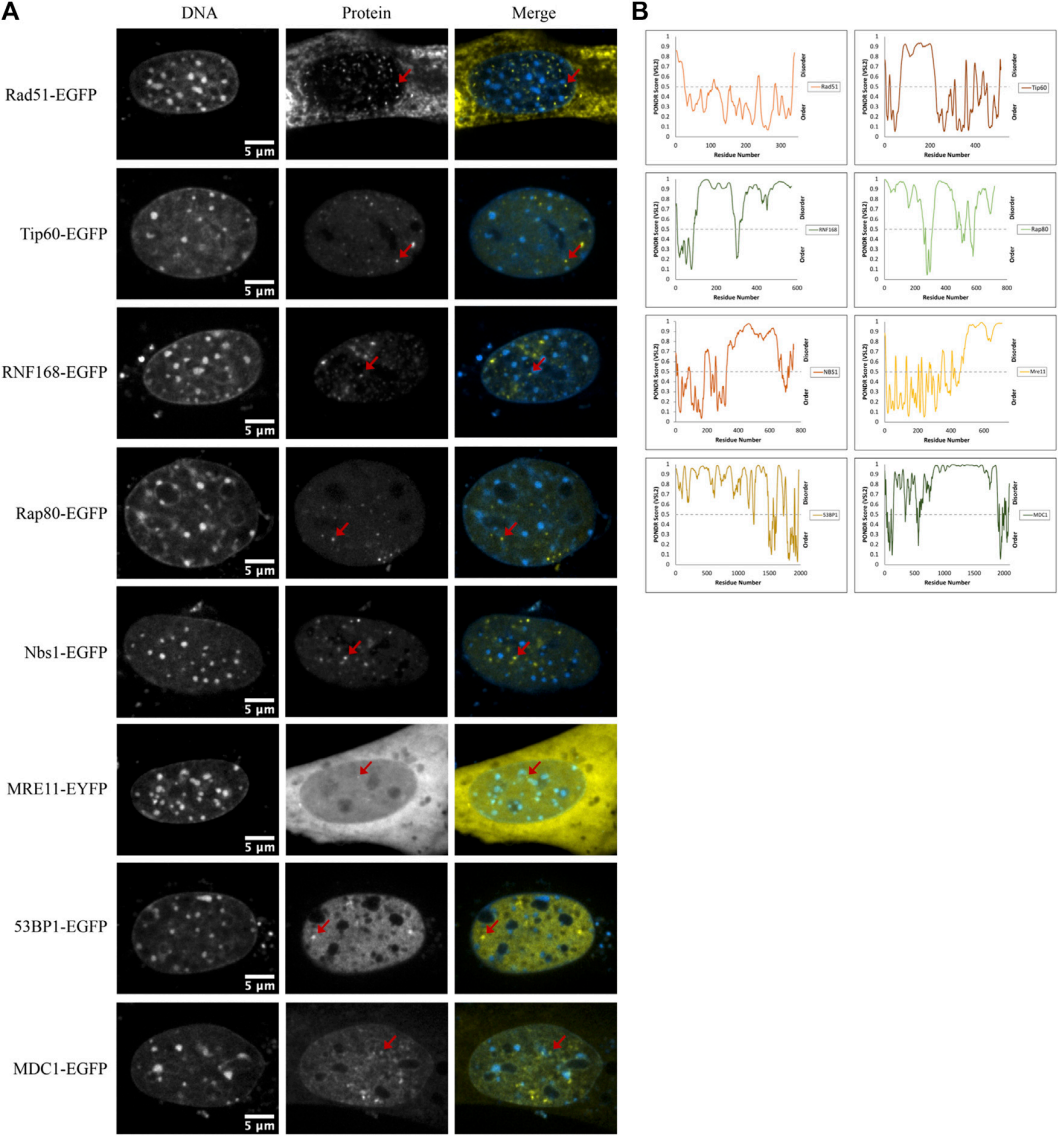
DNA damage sensors, mediators, and effectors form nuclear foci. **(A)** Example images of Rad51-EGFP, Tip60-EGFP, RNF168-EGFP, Rap80-EGFP, NBS1-EGFP, MRE11-YFP, 53BP1-EGFP, and MDC1-EGFP transfected CH3/10T1/2 cells stained with Hoechst 33342 displaying nuclear foci. **(B)** PONDR^®^ scores for each protein predicted by the VSL2 algorithm. A score greater than 0.5 predicts disorder for that region of the protein.

Another mechanism for selectivity independent of size is liquid-liquid phase separation. For example, 53BP1 has been shown to undergo liquid-liquid unmixing to form 53BP1-rich condensates *in vitro*. This reflects a preference for self-interaction over interaction with the solvent (nucleoplasm). Focus formation, particularly upon increased expression, may reflect a potential for differential solubility in distinct solvent environments that are expected to form due to liquid-liquid phase separation. We observed that many of the proteins that we transfected formed nuclear foci. Out of the 17 proteins tested, eight formed nuclear domains that concentrated the fluorescently tagged protein; those eight were Rad51-GFP, Tip60-GFP, RNF168-GFP, Rap80-GFP, NBS1-GFP, MRE11-YFP, 53BP1-GFP, and MDC1-GFP (Figure 7A). Phase separation capacity of proteins is often conferred by regions of disorder (Boeynaems et al., 2018), so to gather a preliminary sense of which proteins may phase separate, we used the program Predictor of Natural Disordered Regions (PONDR^®^) with the VSL2 algorithm to predict disordered regions in proteins that form foci. With the exception of Rad51, each of these proteins contains disordered regions (Figure 7B). Notably, Rad51 does not form foci. Rather, at very low nucleoplasmic concentrations, we find that Rad51 forms short filaments in the nucleoplasm, consistent with previous studies (van der Heijden et al., 2007; Forget & Kowalczykowski, 2010). Foci formation in a subpopulation of cells is expected because of the presence of DNA double-strand breaks even in the absence of external DNA damage sources. However, proteins that form large numbers of small foci are good candidates for forming LLPS condensates. Demonstrating whether or not this reflects phase separation will require future *in vitro* experiments to determine if any of these proteins can initiate liquid unmixing independently.

## Discussion

The condensed state of chromatin in chromocenters has long been thought to contribute to defining the accessibility of molecules. Heterochromatin has increased mutation rates compared to euchromatic regions (Schuster-Böckler & Lehner, 2012). One suggestion to explain this is decreased repair due to lower rates of transcription-coupled nucleotide excision repair (TCR), but another is the attenuated ability of the repair machinery to access heterochromatic regions (Schuster-Böckler & Lehner, 2012; Roberts & Gordenin, 2014). Indeed, H2AX phosphorylation (γH2AX), which is dependent on the large Ataxia Telangiectasia Mutated (ATM) kinase, is reduced in heterochromatic regions in both yeast and mammalian cells (Kim et al., 2007). The concept of chromatin-mediated regulation of accessibility was originally established based on the differential digestion kinetics of active and inactive genes (Weintraub and Groudine, 1976). The association of DNase I sensitivity with the acetylation state of chromatin domains further implicated chromatin folding in regulating genome accessibility (Hebbes et al., 1994) and is consistent with experiments examining the ability of different sized fluorescent dextrans to diffuse into chromatin of differing density (Görisch et al., 2005). Current models propose that chromatin is compacted by interactions with itself (Hansen et al., 2021), and compaction may be further facilitated by proteins and RNA (Thakur et al., 2019; Wang et al., 2019; Fan et al., 2020; Li et al., 2020). Recent studies into the material states of chromatin indicate that chromatin exists in a gel (solid) state (Erdel, 2020; Strickfaden et al., 2020; Hansen et al., 2021). This predicts the existence of pores between crosslinked chromatin fibres. Thus, we might expect that steric hindrance in condensed regions of chromatin could participate in genome regulation. Consistent with this, an inverse relationship between size and localization to constitutive, facultative, and euchromatic regions of mouse nuclei was recently reported, and larger transcriptional complexes were found to be absent in constitutive heterochromatin regions of fixed cells (Miron et al., 2020).

When we examined the distribution of DNA break sensor proteins PARP1, MRE11, and Ku70, we observed variable behaviour for MRE11 and enrichment for both PARP1 and Ku70. In the case of Ku70, the Ku70/80 heterodimer is 11 × 7 × 7 nm and would be expected to have difficulty entering mouse chromocenters if they had a pore size of only 10 nm. PARP1 was significantly enriched in mouse chromocenters. Ku and PARP1 have previously been reported to associate with heterochromatin (el Ramy et al., 2009; Quénet et al., 2008; Song et al., 2001). Accumulation within chromocenters is expected to reflect binding to chromatin within chromocenters, although differential solubility in distinct solvent environments could also explain the enrichment of any of these proteins. In either case, the observed ability of these proteins to diffuse into chromocenters would require that the gel be sufficiently porous to enable these protein complexes to diffuse through their interior. Thus, the pore size must exceed 10 nm diameter.

The case of MRE11 is particularly interesting and, together with the partitioning of NBS1, suggests that the concentrations of diffusing nucleoplasmic proteins are regulated in some manner. MRE11 can be depleted, homogeneous, or enriched within mouse chromocenters. This enrichment was not due to poly(ADP-ribose) as PARP inhibitors did not affect MRE11 distribution. MRE11 forms a complex with NBS1 and Rad50. NBS1 was consistently depleted and to a greater extent than MRE11. This suggests that either the complex is excluded more than MRE11 alone or that these proteins show differential regulation of chromocenter concentration without complex assembly. Importantly, the results indicate that the initial detection of DNA damage within chromocenters is not limited by steric exclusion of sensor proteins since they all show some access to the interior of the mouse chromocenter.

Further supporting the potential physical accessibility barrier to large molecules like ATM, DSB repair in heterochromatin is reported to depend upon chromatin decondensation mediated by the phosphorylation of KAP1 (Goodarzi et al., 2008). Similarly, PARP activation will drive chromatin decondensation, recruitment of chromatin remodelling complexes, and histone displacement (Poirier et al., 1982; Rouleau et al., 2004; Liu & Yu, 2015; Strickfaden et al., 2016). Thus, it may be that some of the mediator and effector proteins involved in DSB repair require chromatin remodelling to increase the porosity of heterochromatin to function there. Work from Tsouroula et al. (2016) establishes that during S/G2, DSBs occurring within chromocenters are relocated to the periphery of chromocenters to undergo repair by HR. In addition, Rad51 assembly occurs on the periphery of chromocenters. In comparison, independent of the cell cycle, DSBs repaired by NHEJ remain within the chromocenter (Tsouroula et al., 2016). Thus, it was of particular interest to assess the ability of ATM and similarly large downstream proteins such as MDC1, 53BP1, and BRCA1 to diffuse into the chromocenter interior. While we did observe that these larger proteins showed greater depletion from mouse chromocenters, in no case did we see evidence for complete exclusion from chromocenters. For example, ATM exists as a dimer in the absence of DNA damage. A monomer of ATM has an approximate height of 20 nm and width of 10 nm (Xiao et al., 2019). The chromocenters are no more than 40% depleted in ATM relative to the surrounding nucleoplasm, with a mean value of 67%. This indicates that significant quantities of even these larger DNA damage response proteins enter chromocenters.

To determine if the movement of the DNA damage response proteins through chromocenters correlated with expected size, we performed fluorescent correlation spectroscopy (FCS) to measure diffusion coefficients across a size range of the proteins. We found that although GFP had an expected small reduction in diffusion within chromocenters. Amongst the other proteins tested, there was not a strong correlation between predicted size and diffusion. For example, 53BP1-EGFP and RNF168-EGFP have the same mean diffusion coefficient in chromocenters. This could reflect the assembly of complexes for RNF168-EGFP. The difference in diffusion coefficient relative to EGFP for 53BP1 is approximately two-fold, while the difference in the predicted size of monomers is approximately 10-fold. Thus, this is close to the expected difference (eight-fold) in mass to account for the reduced diffusion of 53BP1-EGFP relative to EGFP. Nonetheless, 53BP1 diffusion is slightly faster than what we might expect. This is particularly true when considering that the diffusion coefficient measured in the nucleoplasm is slower than in chromocenters. Two possible explanations are that 53BP1 diffuses as a dimer or oligomer in the nucleoplasm but diffuses as monomers through the chromocenters. Purified GFP in permeabilized fixed cells reflected the increased density of the environment with a slower mean diffusion coefficient in the chromocenters compared to the nucleoplasm, as expected because of the higher mass density present in chromocenters (Imai et al., 2017).

One of the most striking features of these results is the wide range in chromocenter concentrations observed for individual proteins. For example, MDC1 and 53BP1 are both depleted from chromocenters. However, we measured concentrations that ranged from 32 to 120% of the nucleoplasmic concentration for MDC1 and from 26 to 67% for 53BP1. For EGFP alone, we observed a range of 53–87% concentration relative to the nucleoplasm. Notably, we found no examples where EGFP was not depleted. However, when we examined fixed and permeabilized cells, we found that purified GFP incubated with permeabilized fixed cells showed near homogeneous distribution between the nucleoplasm and chromocenters. This argues against a volume exclusion effect dictating differences in chromocenter accessibility observed in living cells. That is, the volume occupied by chromatin, and therefore inaccessible to free GFP, is retained during fixation and permeabilization. We expect similar results between living and fixed cells if volume exclusion is responsible for reduced EGFP concentration in the chromocenters of living cells. Another possibility is that the barrier is imposed by a solvent difference arising from the presence of a phase-separated liquid compartment. This barrier would not be expected to be maintained following fixation and detergent extraction. Its removal could explain the failure to maintain a reduced concentration of GFP in the chromocenters of fixed and permeabilized cells. Supporting this interpretation is that the nucleolus is a well-established phase separated compartment in the nucleus (Brangwynne et al., 2011; Feric et al., 2016; Frottin et al., 2019; Lafontaine et al., 2021), and much less depletion was observed for recombinant GFP in the nucleoli of fixed cells relative to the striking depletion in living cells.

Several membraneless compartments in the nucleus are well established as liquid-liquid phase separated (LLPS) condensates, including the nucleolus and nuclear speckles (Boeynaems et al., 2018). Chromocenters are also considered a membraneless compartment, and recent work has established that several key factors in pericentric heterochromatin formation have phase separation capacity *in vitro*. The primary and most explored example of this is heterochromatin protein 1 α (HP1 α), which has been demonstrated to have phase separation capacity *in vitro* (Larson et al., 2017). One current model of heterochromatin formation is that HP1α, which binds H3K9 tri- and dimethylation written by SUV39H1, dimerizes and oligomerizes to bridge nucleosomes compacting chromatin and, at a critical concentration, separates into a heterochromatin phase (Larson et al., 2017). A phase separation role is also supported by work with HP1a in *Drosophila* (Strom et al., 2017). The histone methyltransferase KMT5C that writes H4K20 methylation marks in heterochromatin has liquid-like behaviour in chromocenters in that it exchanges freely within the chromatin but does not exchange freely with the nucleoplasm upon partial bleaching of chromocenters (Strickfaden et al., 2019). Further, another important heterochromatin protein, methyl CpG binding protein 2 (MeCP2), has been suggested to drive heterochromatin condensate formation in association with DNA, and mutations that disrupt the ability of MeCP2 to form condensates *in vitro* are found in patients with the neurodevelopmental disorder Rett syndrome (Li et al., 2020). Work on transcription factor kinetics with single-molecule tracking has recently demonstrated that rather than the previously bi-exponential behaviour with non-specific binding and specific binding, kinetics are better described with a third classification representing IDR-based constraint (Garcia et al., 2021a; Garcia et al., 2021b). It was found that the accumulation of glucocorticoid receptors at distinct regions in the nucleus could not be attributed solely to direct DNA binding events. Rather, there was a second subpopulation reliant on the presence of the transcription factor’s IDR through multivalent interactions consistent with an association with a liquid phase separated compartment (Garcia et al., 2021b). This result supports the idea of the chromocenter as having a liquid phase separated compartment as it supports the accumulation of proteins within the compartment based on their non-specific interaction with other compartment components.

Rad51 forms filaments when overexpressed, and free nucleoplasmic concentrations appear to be kept low because of this propensity to polymerize. Tip60, RNF168, Rap80, and NBS1 all showed the presence of numerous small domains. Interestingly, 53BP1, characterized as a protein capable of initiating liquid-liquid phase separation, formed fewer of these structures. Further studies are required to determine if these are liquid condensates and their concentration dependence. Past work has demonstrated that condensed chromatin behaves as a solid-like gel and may act as a scaffold around which phase separation can occur (Strickfaden et al., 2020) and is supported by the observation that chromatin transitions to a gel-like state upon heterochromatin domain formation during differentiation (Eshghi et al., 2021). In this model, a phase separated condensate would exist around the solid-like gel and would regulate the movement of molecules through the chromocenter (Strickfaden et al., 2020). Differential solubility in a distinct liquid nuclear microenvironment rather than physical exclusion appears to be a better explanation for the relative partitioning of DNA damage response proteins in the nucleoplasm relative to mouse chromocenters. A phase separated condensate model of chromocenters could explain why size alone does not determine the relative abundance of these proteins. In this model, the partition coefficient, reflecting the relative solubility in the two liquid phases, will define the distribution. While this merely changes how the understanding of this distribution should be pursued through mutational analysis, it puts additional demands on the analysis of all proteins that are enriched in liquid compartments. For example, it will be important to distinguish between partitioning through weak multivalent interactions that lead to preferential accumulation in a distinct solvent environment from accumulation mediated by high or low specificity binding to the chromatin, which will also result in accumulation beyond the concentration found freely diffusing within the nucleoplasm.

## Data Availability

The raw data supporting the conclusions of this article will be made available by the authors, without undue reservation.
